# Dideoxynucleoside HIV reverse transcriptase inhibitors and drug-related hepatotoxicity: a case report

**DOI:** 10.1186/1752-1947-1-19

**Published:** 2007-05-08

**Authors:** Giuseppe Lapadula, Ilaria Izzo, Silvia Costarelli, Giuliana Cologni, Luisa Bercich, Salvatore Casari, Marco Gambarotti, Carlo Torti

**Affiliations:** 1 Institute of Infectious and Tropical Diseases, University of Brescia, Italy; 2 Service of Morbid Anatomy, Spedali Civili di Brescia, Italy

## Abstract

This report regards the case of a 43 year-old HIV-positive woman who developed an episode of serious transaminase elevation during stavudine-including antiretroviral therapy. Diagnostic assessment ruled out hepatitis virus co-infection, alcohol abuse besides other possible causes of liver damage. No signs of lactic acidosis were present. Liver biopsy showed portal inflammatory infiltrate, spotty necrosis, vacuoles of macro- and micro-vesicular steatosis, acidophil and foamy hepatocytes degeneration with organelles clumping, poorly formed Mallory bodies and neutrophil granulocytes attraction (satellitosis). A dramatic improvement in liver function tests occurred when stavudine was discontinued and a new antiretroviral regimen with different nucleoside reverse transcriptase inhibitors was used. The importance of considering hepatotoxicity as an adverse event of HAART including stavudine, even in absence of other signs of mitochondrial toxicity should therefore be underlined. Liver biopsy may provide further important information regarding patients with severe transaminase elevation, for a better understanding of the etiology of liver damage.

## Background

Highly active anti-retroviral therapy (HAART) is associated with a number of serious and potentially life-threatening adverse events, including drug-induced liver injury (i.e., hepatotoxicity – HT). The potential of nucleoside reverse transcriptase inhibitors (NRTI) for liver damage seems to be related to mitochondrial DNA damage and can also lead to lactic acidosis [1]. Although *in vitro *data demonstrated a prominent mitochondrial oxidative stress in human hepatoma cells exposed to stavudine [2], and a stronger inhibition of mitochondrial DNA synthesis by dideoxynucleoside analogues (ddX – i.e., stavudine, didanosine and zalcitabine) than by other NRTI [3], existing studies have failed to demonstrate any consistent association between the use of these drugs and the development of subsequent HT [4,5]. Their possible causative role is therefore still under debate.

## Case presentation

**Figure 1:**
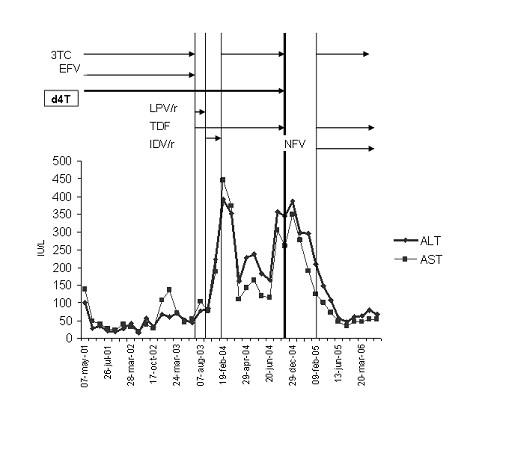
**Liver transaminase and therapeutic evolution in the study patient**. ALT: alanine-amino transferase; AST: aspartate-amino transferase; 3TC: lamivudine; EFV: efavirenz; d4T: stavudine; LPV/r: lopinavir/ritonavir; TDF: tenofovir; IDV/r: indinavir/ritonavir; NFV: nelfinavir.

A 43 year-old woman, HIV positive since 1994 and notified for AIDS in 2001 due to disseminated cytomegalovirus (CMV) infection, underwent routine laboratory testing in February 2004, when a significant increase in alanine amino-transferase (ALT) and aspartate amino-transferase (AST) levels was found (222 and 188 IU/L respectively). Blood re-testing performed after 15 days showed a further increase in transaminase levels (ALT = 392 IU/L, AST = 446 IU/L). The patient was undergoing treatment with stavudine (d4T) 40 mg twice daily, tenofovir (TDF) 300 mg once daily and indinavir 800 mg twice daily, boosted with ritonavir 100 mg twice daily (IDV/r) with poor viro-immunological response (CD4+ T-cell count = 140 cells/mm^3^, HIV-RNA = 1600 copies/ml). The treatment included d4T and TDF since May 2001 and July 2003 respectively, while IDV/r was initiated in October 2003, replacing lopinavir/ritonavir due to gastro-intestinal side effects. (see Fig. 1).

Appropriate investigations were performed to rule out possible causes of liver transaminase elevations. The patient was negative for hepatitis B surface antigen (HBsAg) with a pattern of isolated positive hepatitis B core antibodies (HBcAb). However, plasma HBV-DNA resulted negative. Serum positivity for hepatitis C virus antibodies (HCV-Ab) was reported in May 2001, but chronic HCV infection was excluded by HCV-RNA testing, which resulted undetectable on two consecutive determinations in June and October 2003. Moreover, HCV-RNA was repeated at the time of HT and still resulted negative. Further, hepatitis A virus immunoglobulin M (HAV-IgM) resulted negative and the patient denied any alcohol abuse or concomitant use of other hepatotoxic drugs.

Suspecting a drug-related liver toxicity, IDV/r, the most recently introduced antiretroviral agent, was suspended on February 17^th ^2004 and replaced by lamivudine (3TC). Liver function monitoring and diagnostic assessment were continued.

**Figure 2:**
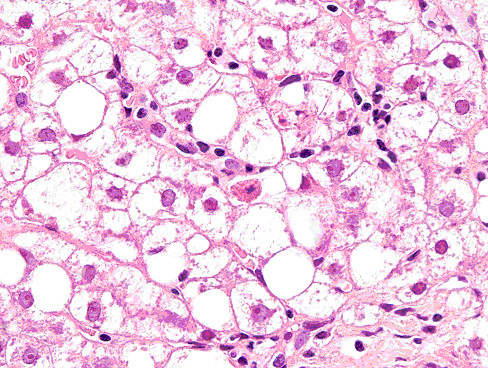
Liver biopsy (ematossilin-eosin 40×) showing acidophil bodies and foamy degeneration of hepatocytes suggestive for a toxic-metabolic disorder.

On March 4^th ^2004, approximatively one month after initial transaminase elevation, ALT was 353 IU/L and AST 373 IU/L. HCV-RNA, HBV-DNA, HAV-IgM resulted negative again, as well as CMV DNA, CMV early-antigen, Epstein-Barr virus sierology and markers indicating auto-immune hepatitis. A venous blood gas sample was obtained, showing pH 7.35, bicarbonate 23 mmol/L, base excess -1.9 mmol/L and plasma lactate 0.6 mmol/L, ruling out lactic acidosis. Ethanol was not detectable in patient serum and no indirect markers of alchol abuse were present. For instance, gamma-glutamil transferase was normal or only mildly elavated, erythrocyte mean corpuscolar volume was within the range of normality during the entire follow-up and AST/ALT ratio did not support an acute alcohol hepatitis. Although the patient presented with lipodistrophy, no signs of metabolic syndrome were present, since triglyceride, cholesterol, fasting glucose and uric acid levels were repeatedly measured and always remained within the range of normality. An abdominal ultrasonography revealed enlargment of the liver, with rounded borders and a bright echopattern, while bilary tract and other intra-abdominal organs were normal. Although a partial improvement of liver function tests followed the therapy switch, three months after IDV/r discontinuation, ALT and AST levels were still elevated (182 and 120/IU, respectively). On May 26^th ^2004, a liver biopsy was performed (see Fig. 2). Histological analysis showed deformation of portal tracts profile due to fibrosis. Portal tracts contained an inflammatory infiltrate of low or focally moderate grade with focal interface hepatitis. Periportal fibrosis was present. Foci of spotty necrosis, acidophil bodies, scattered vacuoles of macro- and micro-vesicular steatosis and scattered lipogranulomas were also present in lobules. Several hepatocytes, predominantly in peri-portal areas, showed ballooning degeneration, clumping of cytoplasm, with the development of poorly-formed Mallory bodies. Ballooned hepatocytes and the poorly formed Mallory bodies were surrounded or infiltrated by neutrophil granulocytes. In addition, scattered hepatocytes had microvacuolated cytoplasma with foamy degeneration. Scattered glycogenated nuclei were present. Immuno-histo-chemical assays excluded the occurrence of an occult HBV infection, showing no reactivity to HBsAg and hepatitis B core antigene (HBcAg) in the liver tissue.

On November 10^th ^2004, antiretroviral drugs were interrupted because of further transaminase elevation and the patient underwent further exams, confirming previous investigations. The patient continued to deny alcohol abuse.

After the interruption of the antiretroviral drugs, liver transaminases decreased, so that, on January 20^th ^2005, a new treatment with tenofovir (TDF), lamivudine (3TC) and nelfinavir (NFV) was initiated due to the lowering of the CD4+ T cells count. Despite the new treatment, between January 2005 and August 2006, the transaminase levels decreased significantly and remained just above the upper limit of normality.

## Discussion

This case suggests a relationship between antiretroviral therapy including d4T and hepatotoxicity. Some authors have suggested that acute liver enzyme elevation in HIV-positive patients is often due to previous conditions, rather than to antiretroviral therapy itself [6]. Specifically, in patients with chronic viral hepatites coexistence, HAART-related HT develops more frequently or sooner, and also in a more severe form [7]. In this group of patients, liver damage may also be caused by immune reconstitution and related exacerbation of viral hepatites [8]. Actual drug-related hepatotoxicities may thus be confounded by the natural history of concomitant viral hepatites in these patients.

*In vitro *studies demonstrated the NRTI are able to inhibit mithocondrial DNA polymerase, DNA polymerase gamma in particular [9]. Moreover, some cases of severe lactate elevation and liver steatosis during HAART including d4T have been reported [10]. In the present case, an important transaminase elevation without lactate elevation was detected. A liver biopsy documented chronic aggressive hepatitis of a possible multifactorial etiology, including initial metabolic disorder with suspected signs of drug hepatotoxicity. Although the histological findings were not specific for drug-induced damage, other causes of liver damage, such as HCV or HBV co-infections, alcohol abuse, metabolic syndrome and assumption of concomitant hepatotoxic drugs were excluded. Therefore, this case report may be particularly relevant since pure drug-induced hepatotoxicity may be postulated, regardless of the "background" effect of chronic hepatitis co-infections. The mechanism of d4T toxicity may have been mediated by liver steatosis, which has already been associated with d4T and other ddX use [11,12]. A steatotic liver, particularly if some grade of fibrosis is present, may be more susceptible to the toxic effect of the drugs. Therefore, use of PI boosted with low dose ritonavir could not be excluded as causative agent of the first episode of transaminase elevation. Although transaminase levels appeared to partially ameliorate after boosted-PI discontinuation, ALT levels always remained at least 3 times higher than the upper limit of normality and a second episode of grade III hepatotoxicity occurred during a NRTI-based regimen. Liver transaminases returned to rather normal values only when NRTIs other than d4T (TDF and 3TC) were administrated. Some authors have observed that switching to alternative NRTIs, such as TDF or abacavir, may reduce lipoatrophy or prevent recurrence of lactic acidosis [13]. Our patient case suggests that a similar phenomenon may occur in the event of hepatotoxicity.

## Conclusion

This case report underlines the importance of d4T as a possible causative agent of hepatotoxicity, even in the absence of other signs of mitochondrial toxicity. Liver biopsy may provide further important information regarding patients with severe transaminase elevation, in order to better assess the possible causative factors.

## Competing interests

The author(s) declare that they have no competing interests.

## Authors' contributions

GL conceived the study and participated in drafting the manuscript. II participated in the study design and in drafting the manuscript. SCL, GC and SC participated in conceiving the study, in the acquisition of data and revised the manuscript. LB and MG analyzed the liver biopsy and revised the manuscript. CT conceived the study and participated in the drafting of the manuscript. All authors read and approved the final manuscript.
